# The Effect of Turmeric (*Curcuma longa*) Extract on the Functionality of the Solute Carrier Protein 22 A4 (SLC22A4) and Interleukin-10 (IL-10) Variants Associated with Inflammatory Bowel Disease

**DOI:** 10.3390/nu6104178

**Published:** 2014-10-13

**Authors:** Mark J. McCann, Sarah Johnston, Kerri Reilly, Xuejing Men, Elaine J. Burgess, Nigel B. Perry, Nicole C. Roy

**Affiliations:** 1 Food Nutrition & Health, Food and Bio-Based Products, AgResearch Grasslands Research Centre, Palmerston North 4442, New Zealand; E-Mails: sljohnston@xtra.co.nz (S.J.); kerri.reilly@agresearch.co.nz (K.R.); xuejing.men@agresearch.co.nz (X.M.); nicole.roy@agresearch.co.nz (N.C.R.); 2 Nutrigenomics New Zealand, Plant and Food Research Ltd., Private Bag 92169, Auckland Mail Centre, Auckland 1142, New Zealand; E-Mails: elaine.burgess@plantandfood.co.nz (E.J.B.); nigel.perry@plantandfood.co.nz (N.B.P.); 3 Gravida: National Centre for Growth and Development, The Liggins Institute, The University of Auckland, Auckland 1142, New Zealand; 4 The Riddet Institute, Massey University, Palmerston North 4442, New Zealand; 5 Department of Chemistry, University of Otago, P.O. Box 56, Dunedin 9016, New Zealand

**Keywords:** turmeric, curcumin, IL-10, inflammatory bowel disease, SLC22A4, OCTN1, nutrigenomics

## Abstract

Inflammatory bowel disease (IBD) is a chronic relapsing disease. Genetic predisposition to the disease reduces an individual’s capacity to respond appropriately to environmental challenges in the intestine leading to inappropriate inflammation. IBD patients often modify their diet to mitigate or reduce the severity of inflammation. Turmeric (*Curcuma longa* L., Zingiberaceae) has historically been used in Chinese, Hindu, and Ayurvedic medicine over several centuries to treat inflammatory disorders. To understand how turmeric may influence the consequences of a genetic predisposition to inappropriate inflammation, we used HEK293 cells to examine the *in vitro* capacity of turmeric extract and fractions to affect the functionality of two gene variants, solute carrier protein 22 A4 (SLC22A4, rs1050152) and interleukin-10 (IL-10, rs1800896) associated with IBD. We found that a turmeric extract and several chromatographically separated fractions beneficially affected the variants of SLC22A4 and IL-10 associated with IBD, by reducing inappropriate epithelial cell transport (SLC22A4, 503F) and increasing anti-inflammatory cytokine gene promoter activity (IL-10, −1082A). The effect of turmeric on the IL-10 variant was strongly associated with the curcumin content of the extract and its fractions.

## 1. Introduction

Inflammatory bowel disease (IBD), classified as ulcerative colitis (UC) or Crohn’s disease (CD), is a chronic relapsing disease that is characterised by an inappropriate immune response by the intestinal mucosa [1,2]. Whilst the precise aetiology of IBD remains to be established, a genetic predisposition to an inappropriate immune response and/or impaired barrier function of the gastrointestinal tract (GIT) and how the mucosa subsequently respond to environmental challenges, such as diet and microbiota, is considered to be central in understanding how to ameliorate the severity of the disease [3,4,5,6]. Epidemiological, familial, and genome-wide association studies have shown that the risk and severity of IBD are based on multiple and inter-related gene alterations. These compromise an individual’s capacity to respond appropriately to environmental challenges in the GIT leading to chronic inflammation [7,8,9].

Turmeric (*Curcuma longa* L., Zingiberaceae) is a food spice and colouring agent used in Chinese, Hindu, and Ayurvedic medicine over several centuries to treat a wide range of diseases including inflammatory disorders [10,11,12]. Its characteristic yellow-orange colour is due to curcuminoids, which are compounds that have been linked with anti-inflammatory effects based on mediating cell signalling pathways, expression of genes encoding inflammatory cytokines, growth factors, enzymes and cell cycle proteins, or through direct interaction with multiple molecular targets [10,11,13,14,15,16]. However, it is unclear if turmeric spice or its curcuminoid components can affect the function of gene variants associated with IBD.

There are several interdependent molecular pathways involved in IBD pathogenesis, including intestinal epithelial barrier function and immune response [17]. The solute carrier family 22 member 4 (SLC22A4, commonly known as OCTN1) gene codes for an organic cation transporter protein spanning the plasma membrane of epithelial cells. The risk variant (rs1050152) of SLC22A4 for IBD occurs in exon 9 where a cytosine is substituted with a thymidine at position 1507 of the coding sequence, resulting in a phenylalanine (F) replacement of the normal leucine (L) amino acid at position 503 of the SLC22A4 protein [18]. The 503F variant has a higher transport activity than the 503L variant and the resulting inappropriate transport of organic cations across the intestinal epithelial barrier is thought to contribute to IBD pathogenicity [18,19,20,21,22,23,24,25,26].

Interleukin-10 (IL-10) is an immune-suppressive cytokine that acts after the initial inflammatory response to repress excessive pro-inflammatory cytokine activity [27]. Insufficient production of IL-10 is thought to create an imbalance between pro- and anti-inflammatory mechanisms and several studies show that this affects IBD severity [28,29,30,31,32,33,34,35,36,37]. One of the risk variants of IL-10 associated with IBD is the rs1800896 single nucleotide polymorphism in the promoter region of the IL-10 gene, where an adenine (A) substitution for a guanine (G) at position −1082. The −1082A variant has been linked with lower IL-10 transcription and cytokine production in IBD and may explain, in part, the inappropriate inflammatory response observed in the disease [38,39].

We hypothesised that turmeric affects the inappropriate function of gene variants associated with IBD and to investigate this we examined the capacity of turmeric extract and fractions to affect the abnormal function of the SLC22A4 variant, 503F, and the IL-10 promoter variant, −1082A, in HEK293 cells transfected with these genes. Our screening assays were designed to identify potential bio-activity in the IBD-associated variants prior to studies *in vivo*. To examine if any turmeric bio-activity was related to curcuminoids, the curcumin content of the extract and fractions were measured by HPLC.

## 2. Experimental Section

### 2.1. Cell Culture

Authenticated Flp-In™ 293 (Flp293) and 293/TLR4-MD2-CD14 (293TLR4) cell lines were purchased from the Life Technologies (Auckland, New Zealand) and Invivogen (San Diego, CA, USA), respectively. All cell culture reagents were obtained from Gibco (Life Technologies, New Zealand) unless otherwise stated. The Flp293 and 293TLR4 cell lines were cultured in high glucose Dulbecco’s Modified Eagle Medium (DMEM) supplemented with 10% foetal bovine serum, 1% PenStrep, and 100 µg/mL of Normocin (293TLR4 only, Invivogen, USA). Selective pressure was maintained using 100 g/mL of Zeocin™ (Flp293 cells) or 10 μg/mL of Blasticidin and 50 μg/mL of HygroGold™ (293/TLR4 cells, Invivogen, USA). Antibiotic-free medium was used for the SLC22A4 and IL-10 assays.

### 2.2. Chemicals

All chemicals were purchased from Sigma-Aldrich (Auckland, New Zealand) unless otherwise stated. For the SLC22A4 assay, a transport buffer was prepared as described previously [18]. A stock solution of methyl ^14^C-betaine (18.33 MBq, ARC Inc., St. Louis, MO, USA) was used to prepare a working stock (1032 Bq) for the transport assay. L-(+)-ergothioneine (ET, E7521, Sigma-Aldrich, Auckland, New Zealand) was used at 100 nM as a positive control for the SLC22A4 assay. For the IL-10 assay, ultra-pure lipopolysaccharide (LPS, Invivogen, USA) was used at 1 μg/mL for TLR4-specific induction. Dexamethasone (DEXA, D4902, Sigma-Aldrich, New Zealand) was used at 1 μM as a positive control for LPS-mediated IL-10 induction [40].

Curcumin (C8850/43, Acros Organics, Geel, Belgium) was used to calibrate the HPLC analyses of the turmeric extract and fractions. Etoposide (ETO, E1383, Sigma-Aldrich, New Zealand) was used as a positive control for the solvent tolerance assay.

### 2.3. Preparation of Ethanol Extracts and Reversed-Phase Fractions from Turmeric

Solvents of high-performance liquid chromatography (HPLC) grade were obtained from Thermo Fisher Scientific (Scoresby, Melbourne, Australia). Ten grams of commercially-available turmeric spice was extracted with 100 mL of ethanol (EtOH)/H_2_0 (96:4, v/v) overnight by shaking. The ethanol extract was filtered prior to fractionation using a 5 g C18 Isolute solid-phase extraction (SPE) cartridge conditioned with 10 mL of EtOH followed with 10 mL of 1:1 EtOH/H_2_O, and finally with 10 mL of H_2_O. An aliquot of extract (50 mL) was coated onto 5 g C18 (Aldrich octadecyl-functionalised silica gel) by rotary evaporation at 30 °C and applied to the pre-conditioned SPE cartridge [41]. This was eluted with 2 × 10 mL each of H_2_O, 1:4 EtOH/H_2_O, 1:1 EtOH/H_2_O, 4:1 EtOH/H_2_O, EtOH, and ethyl acetate to give twelve 10 mL fractions, which were collected into glass tubes.

Aliquots (1 mL) of extract and fractions were dried at 20 °C in Eppendorf safelock tubes and stored at −20 °C until required. Prior to each assay, the aliquots of turmeric extract (E) and fractions 6–12 were resuspended in 250 μL of dimethyl sulfoxide (DMSO) and vortexed twice for 30 s. Due to the different polarity of the fractions, fractions 1–6 were resuspended in 250 μL of DMSO and 750 μL of sterile UltraPure water (Life Technologies, New Zealand) and then vortexed twice for 30 s.

### 2.4. Quantification of Curcumin Content of Turmeric Extract and Fractions

HPLC analyses of the curcumin content of the turmeric extract and fractions was completed using an Agilent 1200 HPLC, controlled with EziChrom Elite software (Agilent, Santa Clara, CA, USA), at 20 °C on a C18 column (Phenomenex Luna ODS(3) 5 μm 100 A 150 × 3 mm, (Phenomenex, Auckland, New Zealand) with a 2 × 4 mm C18 guard column. Peaks were detected at 210, 254, and 280 nm. The mobile phase used was (A) acetonitrile with 0.1% CH_2_O_2_ (formic acid) and (B) H_2_O with 0.1% formic acid, at the ratio of A:B with run time as *t*_0 min_ = 10%, *t*_12.5_ = 100%, *t*_15_ = 100%, *t*_16_ = 10%, *t*_20_ = 10%. The flow rate was 0.5 mL/min, with an injection volume of 5 μL. Under these conditions curcumin eluted after 12.25 min.

### 2.5. Solvent Tolerance of HEK-293 Cells

The effect of DMSO on the metabolic activity of the cell lines after 24 h was measured using the water-soluble tetrazolium (WST-1) cytotoxicity assay (Clontech, Mountain View, CA, USA) [42,43].

The tolerance of the cells to the solvent DMSO (0%, 0.06%, 0.13%, 0.25%, 0.50%, 1.0%, and 2.0% (v/v)) was tested on eight replicates of 9 × 10^3^ cells per well in 96-well tissue-culture plates (Corning, Edison, NJ, USA), as described previously [43]. Etoposide at 20 μM was used as a positive control [44].

### 2.6. Expression of SLC22A4 rs1050152 Variants in Flp293 Cells and SLC22A4 Transporter Assay

The Flp-In™ system (Invitrogen, Auckland, New Zealand) was used for stable transporter expression as described previously [18]. The transport capacity of Flp293 cells stably transfected with either SLC22A4-503L (503L) or the SLC22A4-503F (503F) variants was based on the uptake of methyl ^14^C-betaine by the cells and quantified by liquid scintillation counting as described previously [18]. The assay was completed as 24 or 12 independent replicates for ergothioneine or turmeric screening, respectively.

### 2.7. Expression of IL-10 rs1800896 Variants in 293TLR4 Cells and IL-10 Promoter Activity Assay

The IL10 promoter variants were prepared by amplifying the IL10 promoter region (−1149 to +31) and the −1082 from G to A mutated using GeneArt site directed mutagenesis kit (Life Technologies, New Zealand). The 293TL4 cells were transiently transfected with a pMetLUC2 reporter plasmid (Clontech, USA) containing the IL-10 −1082A variant and the pSEAP2 control vector to normalise for transfection efficiency using the Lipofectamine^®^ LTX with Plus™ system (Life Technologies, New Zealand) according to the manufacturer’s protocols.

For each IL-10 promoter variant, 1.5 × 10^5^ cells were transiently co-transfected with 500 ng each of pMetLUC2-IL-10(GCC) or pMetLUC2-IL-10(IBD-associated, ACC) and pSEAP2 in Opti-MEM prior to seeding into tissue-culture grade 96-well plates (Corning, USA) and incubated at 37 °C with 5% CO_2_ in a humidified atmosphere for 24 h to facilitate attachment. The medium in each well was then replaced with either medium-only (negative control) or test solutions (in medium). IL-10 transcription was induced by adding 1 μg/mL of LPS to each sample and incubation at 37 °C with 5% CO_2_ in a humidified atmosphere for 2 h. After 2 h, two aliquots of 50 μL of each sample were transferred to separate opaque 96-well plates (Corning, USA). The secreted luciferase in one set of samples was measured using the Ready to Glow luciferase kit (Clontech, USA) according to the manufacturer’s protocols. The remaining set of samples was measured for secreted alkaline phosphatase using the same kit according to the manufacturer’s protocols.

The luminescence of both samples was measured using a FlexStation 3 multi-mode plate reader (Molecular Devices, Sunnyvale, CA, USA). The assay was completed as twenty-four or three independent replicates for dexamethasone or turmeric screening, respectively.

### 2.8. Statistical Analyses

All data were analysed for statistical significance using a one-way ANOVA with SigmaStat 12.3 (Systat Software Inc., San Jose, CA, USA). The normality of the data was tested using the Shapiro-Wilk method and the equality of variance using the Leven Median test. Non-normally distributed data was ranked and analysed using the Kruskal-Wallis ANOVA method. Following ANOVA, significantly different means were identified using the Dunnett’s *post-hoc* test. A probability (*p*) value of less than 0.05 was considered to show a significant difference.

## 3. Results

### 3.1. Curcumin Content of Turmeric Extract and Fractions

**Figure 1:**
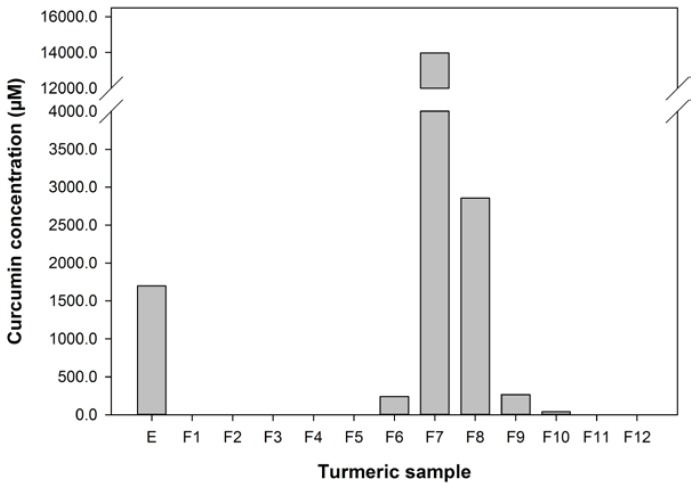
The concentration of curcumin in the turmeric samples used in this study.

The curcumin content of the turmeric used, extracted into ethanol, was about 0.6% w/w of the dry spice, which is at the low end of the range of curcumin contents found for turmeric from various sources [45]. The extract was fractionated to give 12 fractions ranging from water soluble in F1 to lipids in F12. HPLC analyses of the extract and fractions (Figure 1) showed that this fractionation protocol was effective at concentrating curcumin in fractions 7 and 8, eluted with 4:1 EtOH/H_2_O.

### 3.2. Solvent Tolerance of HEK293 Cells

To ensure that any bio-activity in the HEK293 cell types was due to the sample and not the DMSO solvent used the effects of 0.05 to 1% DMSO on the metabolic activity of the cell line were measured using the WST-1 assay. The amount of DMSO solvent used in the gene-specific assays, 0.25% (v/v), did not affect the innate metabolic activity of the HEK293 cell types. The positive control, 20 μM etoposide (ETO), inhibited metabolic activity as expected (*p* < 0.05).

### 3.3. Turmeric Reduces the Abnormal Transport of the IBD-Associated SLC22A4 Variant

The uptake of ^14^C-betaine by HEK293 cells transfected with the SLC22A4-503F variant (91.2 ± 7.0 Bq) was significantly higher than that of the 503L variant (50.2 ± 5.9 Bq) (*p* = 0.001). This is in agreement with previous work [18,23]. The uptake of ^14^C-betaine by ET-treated 503F variant cells was reduced to that of untreated 503L variant cells (503F:51.1 ± 7.5 *vs.* 503L:50.2 ± 5.9 Bq).Therefore our assay was an appropriate model to study how the higher transport functionality of 503F variant may be reduced to a more normal level by food compounds. Furthermore the reduction in transport measured in response to ET by the 503F variant shows that our assay can be used to assess how a dietary compound affects the abnormally high transport activity this variant.

**Figure 2:**
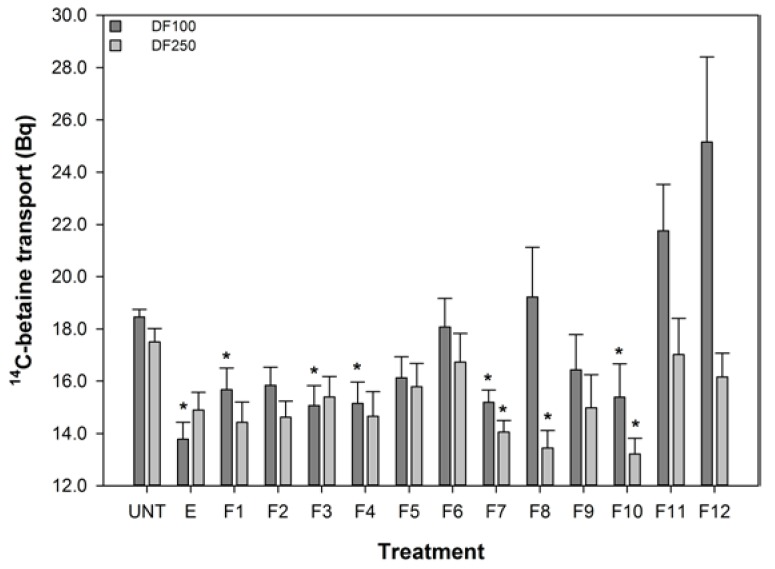
The effect of turmeric on ^14^C-Betaine transport by the SLC22A4 503F variant. DF refers to dilution factor and a value lower than the untreated (UNT) indicates reduced transport. The data are expressed as the mean (±SEM) of twelve independent replicates. A statistical difference between untreated and treated cells is indicated by * *p* < 0.05.

The effect of turmeric extract and fractions, at 1 in 100 and 1 in 250 dilutions of the reconstituted aliquots, on the uptake of ^14^C-betaine by the 503F variant were measured and these data are shown in Figure 2. These dilutions were chosen because pure curcumin was only effective at reducing ^14^C-betaine uptake in the 503F variant at these dilutions (data not shown). These data show that a 1 in 100 dilution of turmeric extract and fractions 1, 3, 4, 7, and 10 significantly reduced the transport of methyl ^14^C-betaine relative to untreated 503F variant cells. LC analyses (Figure 1) showed that curcumin was concentrated in active fraction 7, but not in the other fractions active in this assay. Therefore curcumin is not the main transport inhibitor, and unidentified hydrophilic (fractions 1–4) and lipophilic (fraction 10) components are also active. There may be synergistic effects, since the concentrated fractions are not significantly more active than the extract (Figure 2).

The turmeric extract and fractions 1, 3, 4, 7, and 10 significantly reduced betaine transport in the 503F variant at 1 in 100 dilution. Only fractions 7, 8, and 10 were effective at the 1 in 250 dilution. Interestingly, both the 1 in 100 and 1 in 250 dilutions inhibit the transport of ^14^C-betaine, compared to untreated cells, by a similar amount despite the substantial difference in curcumin content (28.6 *vs.* 11.4 μM). However, the curcumin content of the turmeric samples (Figure 1) indicates that although the extract and fractions 7 and 8, in particular, have high level levels of curcumin, this did not satisfactorily account for the activity of the more water-soluble fractions 1, 3, and 4.

These data show that the reduced transport function of the 503F variant does not correlate with curcumin concentrations, especially the reductions in transport by the more water soluble fractions (1–4) with no detectable curcumin content.

### 3.4. Turmeric Increases the Promoter Functionality of the IL-10 Variant Associated with IBD

**Figure 3:**
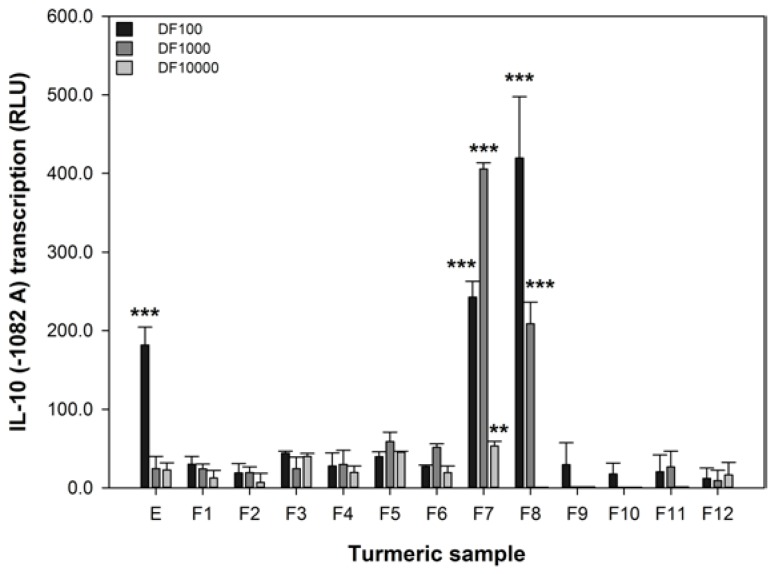
The effect of turmeric on the transcription of the IL-10 −1082 A variant. RLU refers to relative luminescence units and a positive value indicates an increase in promoter transcription. The data are expressed as the mean difference (±SEM) of three independent replicates compared to the untreated sample. A significant difference between untreated and treated cells indicated by ** *p* < 0.01, or *** *p* < 0.001.

The IL-10 promoter activity of 293/TLR4-MD2-CD14 cells transiently transfected with the -1082 A variant was significantly reduced by 1 μM DEXA (reduced from 5.8 ± 3.3 to 1.7 ± 2.4, *p* = 0.012). The luciferase activity of IL-10 (−1082 A) promoter variant can be modified in our assay by DEXA, indicating that our assay is responsive to a known anti-inflammatory agent [40]. The effect of turmeric extract and fractions on the transcription of the IL-10 promoter variant associated with IBD, −1082 A, was measured and these data are shown in Figure 3. These data show that only the extract and the fractions with high levels of curcumin (Figure 1) were effective at increasing the transcription of the −1082 A variant. At the 1 in 100 dilution, the extract and fractions 7 and 8 were active. However, at the 1 in 1000 dilution only fractions 7 and 8 remained active and fraction 7, in particular, was more effective. Only fraction 7 was active at the 1 in 10,000 dilution. These data are interesting as they show that the activity of turmeric in the IL-10 assay is strongly associated with curcumin content. Furthermore, curcumin is effective at increasing the reduced IL-10 −1082 A transcription associated with IBD at 140, 14, and 1.4 μM (fraction 7).

There is a strong association between the curcumin content of turmeric in both of the concentrated fractions F7 and F8 and their capacity to increase the activity of IL-10 −1082 A variant.

## 4. Discussion

This study is, to our knowledge, the first to directly assess the effect of turmeric on the function of two gene variants associated with IBD. In this study we have developed two gene-specific *in vitro* assays to examine the capacity of food to affect variants of SLC22A4 and IL-10 that are associated with IBD and validated these with responses with known active compounds (ET and DEXA, respectively) [18,40]. We used HEK293 cells as our *in vitro* model as although an intestinal cell line may provide more authentic data, the currently available human intestinal cell lines are derived from adenocarcinomas and may confound our findings. Additionally, HEK293 cells are easy to transfect and are widely used for recombinant protein studies [46]. We have shown that turmeric and specific fractions of it can: (1) reduce the abnormal transport function of the SLC22A4 503F variant and (2) increase the activity of the IL-10 promoter variant (ACC, −1082 A) that has reduced activity in IBD.

These assays allow screening for food compounds that may affect two important and inter-related pathways that are thought to function inappropriately in IBD, intestinal epithelial transport of molecules and the regulation of the immune response [17,18,27,29]. Inappropriate transport of molecules by epithelial cells in the GIT can lead to cellular dysfunction and may trigger an inappropriate inflammatory response [17,22]. Our data have shown that the turmeric extract and some fractions can reduce the abnormal higher transport of compounds reported in the SLC22A4 503F variant [18,23,24]. These reductions may reduce inappropriate triggering of an inflammatory response, although the reduction in transport activity was weak and may not be biologically meaningful.

The appropriate regulation of the inflammatory response, whether or not it is due to altered epithelial cell transport, is central to IBD pathogenesis and severity [27,28,29]. IL-10 functions by dampening the initial pro-inflammatory response, and if this dampening is compromised due to reduced IL-10 transcription, for example, then when an immune response occurs it may last longer than is appropriate resulting in an excessive immune response that can worsen the severity of IBD [17,33,36,38]. Our data have also shown that the abnormal reduction in the activity of the IL-10 −1082 A variant can be improved by the turmeric extract and some fractions. This increase in transcription activity may lead to a more normal level of IL-10 transcription and subsequent anti-inflammatory activity. However, this would require further validation at the protein level using western blotting or an ELISA assay. Our method only assesses the effect on the transcription of the promoter sequence of the IL-10 gene and not the full IL-10 gene and subsequent protein abundance and activity.

Turmeric contains high concentrations of curcumin [12,13,14,45] and we have shown that curcumin concentration correlates with increase the activity of IL-10 −1082 A variant (Figure 1 and Figure 3). However, it must be noted that the effective concentrations of the extract and enriched fractions in this *in vitro* assay, 140, 14, and 1.4 μM, are much higher than the 0.06 μM achieved in human plasma using high doses of a specially formulated curcumin [47]. However, the local concentration of curcumin in the gut might be far higher than the 0.06 μM in the plasma and therefore a beneficial effect on the intestinal epithelium may occur at the effective concentrations found in this study. Given the responses shown in Figure 2, it seems that no single turmeric component was predominantly responsible for capacity of turmeric extract to influence transport of methyl ^14^C-betaine relative to untreated 503F variant cells.

Although our data show that turmeric can beneficially affect IBD-associated gene variants, we have not shown how turmeric affects the wild-type variants of these genes, or that for example increased IL-10 promoter expression results in increased IL-10 production and secretion. Curcumin is known to have several anti-inflammatory effects [10,11,13,14,15,16] and it is therefore likely that IBD patients (and those suffering from other gut diseases) may benefit from curcumin independently of the two gene variants we studied. It is also plausible that other IBD-associated gene variants and/or the complex interactions actions between the known IBD-associated variants could be affected by curcumin; however this was not the focus of the current paper (although it is part of the Nutrigenomics New Zealand programme). Our assays represent the initial screening step for potential bio-activity of food compounds, and focused on identifying the effect on the biologically-relevant variants associated with IBD, prior to more detailed functional studies in more complex biological models. We have shown that our approach is valid for initially screening and more importantly measuring the effects of food compounds in key gene variants associated with IBD.

## 5. Conclusions

IBD is a chronic relapsing incurable disease that occurs due to the inappropriate activity of several genes involved in normal intestinal cell function and the immune response. People with IBD tolerate foods differently and dietary management of the disease is a frequent occurrence. If diet affects the functional consequences of a genetic predisposition to IBD, it is essential that the interactions between food and gene variants associated with IBD are studied in order to better understand how the severity of the disease can be managed. We have shown *in vitro* that turmeric, partly due to its curcumin content, can beneficially affect two gene variants linked to IBD severity. Given the relative ease of adding turmeric to a diet, IBD sufferers with the gene variants tested may benefit from increased turmeric consumption, subject to further *in vivo* and proof of efficacy in human studies.
